# Supplementary material for: Bile acids activate the antibacterial T6SS1 in the gut pathogen Vibrio parahaemolyticus
Source: Microbiol Spectr. 2024 Aug 20;12(10):e01181-24. doi: 10.1128/spectrum.01181-24 (PMC11448226; doi:10.1128/spectrum.01181-24)
Supplement: Supplemental materials — Supplemental methods, figures, tables, and references. [file spectrum.01181-24-s0001.docx]

**Supplementary Information**

**Bile acids activate the antibacterial T6SS1 in the gut pathogen *Vibrio parahaemolyticus***

Supplementary Materials and Methods

Supplementary Figures S1-S2

Supplementary Tables S1-S2

Supplementary References

**Supplementary Materials and Methods**

**Strains and media:** Bacterial strains used in this study are listed in **Table S1**. *Escherichia coli* strains were grown in 2xYT broth (1.6% [wt/vol] tryptone, 1% [wt/vol] yeast extract, and 0.5% [wt/vol] NaCl) or on Lysogeny broth (LB) agar plates (1% [wt/vol] tryptone, 0.5% [wt/vol] yeast extract, 1% [wt/vol] NaCl, and 1.5% [wt/vol] agar) at 37°C. Media were supplemented with kanamycin (30 μg/mL) or chloramphenicol (10 μg/mL) when appropriate to maintain plasmids. *Vibrio parahaemolyticus* (*Vpara*) and *Vibrio natriegens* strains were routinely grown in MLB (LB containing 3% [wt/vol] NaCl) or on marine minimal media (MMM) agar plates (2% [wt/vol] NaCl, 0.4% [wt/vol] galactose, 5 mM MgSO_4_, 7 mM K_2_SO_4_, 77 mM K_2_HPO_4_, 35 mM KH_2_PO_4_, 2 mM NH_4_Cl, and 1.5% [wt/vol] agar) at 30°C. Media were supplemented with kanamycin (250 μg/mL) or chloramphenicol (10 μg/mL) when appropriate to maintain plasmids. For secretion assays, fluorescence reporter assays, and growth assays, *Vpara* were grown in M9 media (1X M9 minimal salts base [FORMEDIUM, MMS0102], 0.8 mM MgSO_4_, 5 μM CaCl_2_, 0.4% [wt/vol] glucose, and supplemented with 3% [wt/vol] NaCl).

**Plasmid construction:** Plasmids used in this study are listed in **Table S2**. To construct transcription reporter plasmids, 300 bp upstream of the *vp1400* or 200 bp upstream of the *vpa1270* open reading frames were amplified from the genome of *Vpara* RIMD 2210633 and introduced into the multiple cloning site of pVSV33 (1), upstream of a promoterless *cat* and *gfp* reporter cassette, using the Gibson assembly method (2).

**Construction of deletion strains:** To delete *tfoY* (*vp1028*) in *Vpara* RIMD 2210633, *E. coli* DH5α λ-pir cells containing the pDM4:*tfoY* plasmid were conjugated into *Vpara* RIMD 2210633. Transconjugants were selected on MMM agar supplemented with chloramphenicol. The resulting transconjugants were plated onto MMM agar containing 15% (wt/vol) sucrose for counterselection and loss of the sacB-containing pDM4. Deletions were confirmed by PCR.

**Fluorescence reporter assays and growth assays:** Overnight‐grown cultures of *Vpara* strains containing the indicated pVSV33-based plasmids were diluted 1:10 in fresh MLB supplemented with kanamycin to maintain the plasmids, and grown at 30°C for two hours. Then, the cultures were normalized to an OD_600_ = 0.1 in M9 media supplemented with 3% (wt/vol) NaCl and kanamycin, and with either 20 μM phenamil (TOCRIS, 3379; 1 mM stock solution prepared in 20% [vol/vol] DMSO), 20% (vol/vol) DMSO in volumes equivalent to those added for the phenamil treatment, or the indicated final concentrations of sodium deoxycholate (DOC; Sigma, 309710) or sodium taurodeoxycholate hydrate (TDC; Sigma, T0557). The normalized samples were transferred to black, sterile, flat-bottom 96‐well microplates (Greiner, 655090) in triplicate (200 μL per well) and grown at 30°C in a BioTek SYNERGY H1 microplate reader with continuous shaking (205 cpm). Every 15 minutes, cell density readings (OD_600_) and fluorescence readings (excitation 479 nm, bandwidth 18 nm; emission 520 nm, bandwidth 18 nm) were taken. GFP reporter activity was analyzed as arbitrary units obtained by dividing the fluorescence reading by the OD_600_ reading after subtracting blank media readings. The experiments were performed at least three times with similar results. Results from a representative experiment are shown.

**Chloramphenicol-resistance reporter assays:** *Vpara* strains containing the indicated pVSV33-based plasmids were streaked onto MLB agar plates supplemented with kanamycin to maintain the plasmids, or kanamycin and chloramphenicol. Plates were incubated overnight at 30°C, and *cat* reporter expression was determined as growth on plates containing chloramphenicol.

**Secretion assays:** *Vpara* strains were grown in MLB at 30°C overnight. Overnight bacterial cultures were diluted 1:10 in fresh MLB and grown at 30°C for two additional hours. Then, bacterial cultures were normalized to OD_600_ = 0.18 in M9 media supplemented with 3% (wt/vol) NaCl and incubated for four hours at 30°C in the presence or absence of 20 μM phenamil, 20% (vol/vol) DMSO in volumes equivalent to those added for the phenamil treatment, or 0.025% (wt/vol) DOC. For expression fractions (cells), 0.5 OD_600_ units of cells were harvested and re‐suspended in 50 μL of 2X Tris‐glycine SDS sample buffer (Novex, Life Sciences) supplemented with 5% (vol/vol) β-mercaptoethanol. Supernatants of volumes equivalent to 5 OD_600_ units were filtered (0.22 μm) and precipitated with deoxycholate and trichloroacetic acid (3). Precipitated proteins were washed twice with ice‐cold acetone prior to re‐suspension in 20 μL of 100 mM Tris–HCl pH = 8.0, followed by the addition of 20 μL of 2X Tris‐glycine SDS sample buffer supplemented with 5% (vol/vol) β-mercaptoethanol. Expression and secretion samples were resolved on TGX stain‐free gels (Bio‐Rad), transferred onto nitrocellulose membranes, and immunoblotted with custom‐made α‐VgrG1 (for T6SS1) (4) at a 1:1000 dilution, and α-RpoB (Direct-blot^TM^ HRP anti-*E. coli* RNA Polymerase β antibody; BioLegend, 663907). Results of a representative experiment out of three independent experiments are shown.

**Competition assays:** Bacterial competition assays were performed as previously described (5), with minor modifications. *Vibrio natriegens* prey strains containing pBAD33.1 plasmids for chloramphenicol selection were grown for 16 hours in MLB media supplemented with chloramphenicol at 30°C; *Vpara* attacker strains were grown for 16 hours in M9 media supplemented with 3% (wt/vol) NaCl at 30°C. In the morning, *Vpara* attacker strains were diluted 1:4 into fresh media and divided into two samples, one of which was supplemented with DOC to a final concentration of 0.025% (wt/vol). The *Vpara* cultures were grown for two additional hours at 30°C and then washed with 1 mL of MLB medium. Next, attacker and prey cultures were normalized to an OD_600_ of 0.5 and mixed at a 4∶1 (attacker:prey) ratio in triplicate, and the mixtures were spotted (25 μL) on MLB agar competition plates and incubated at 30°C for two hours. The colony-forming units (CFU) of the prey strains at t = 0 h were determined by plating tenfold serial dilutions on selective media plates. After two hours of co-incubation on competition plates, the bacteria were harvested, and the CFUs of the surviving prey strains were determined as described above. Prey strains contained a pBAD33.1 plasmid to allow selective growth on plates containing chloramphenicol.

**
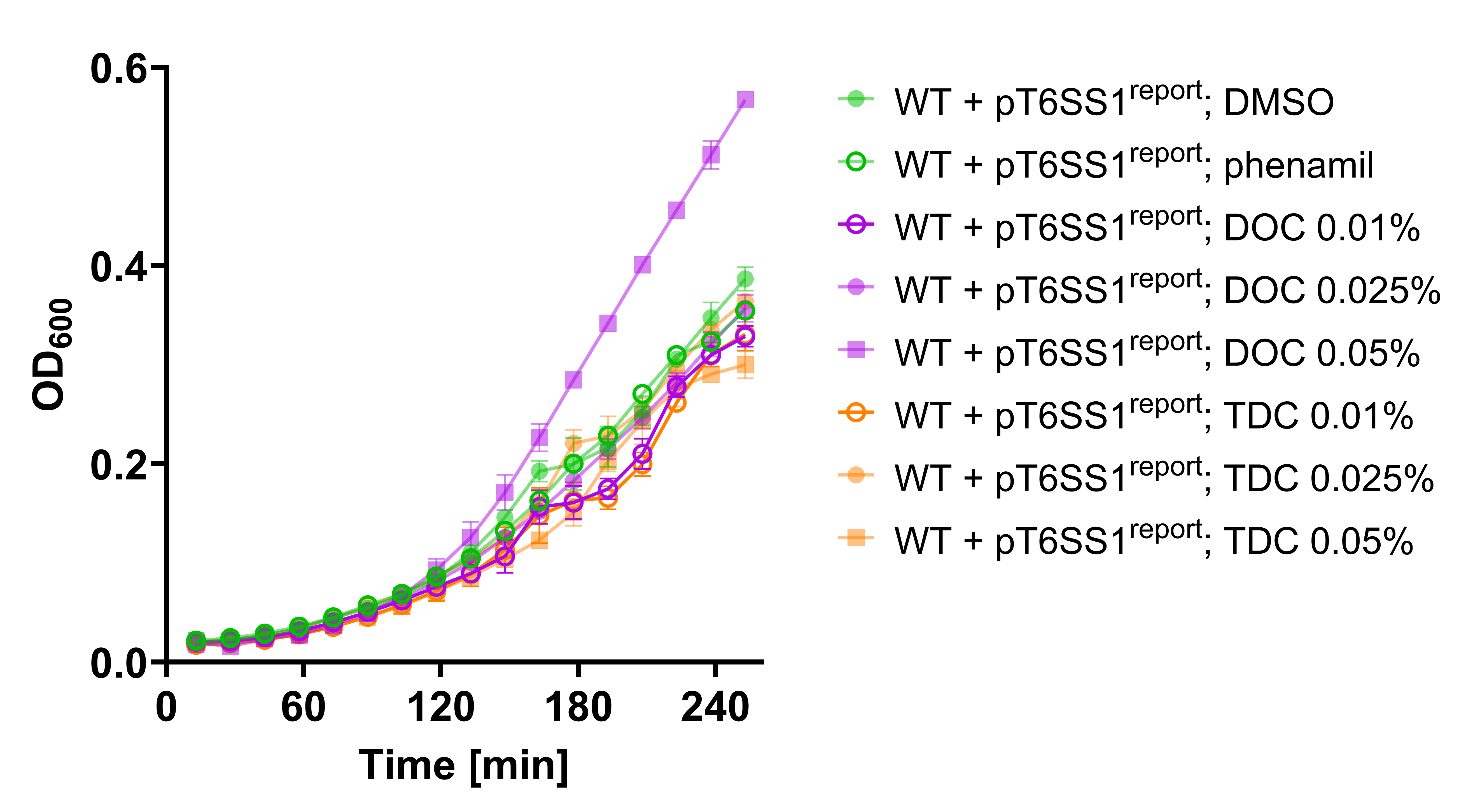
Supplementary Figures**

**Fig. S1. Bile acids do not hamper *Vibrio parahaemolyticus* growth.** Growth of *V. parahaemolyticus* RIMD 2210633 carrying a plasmid containing a *gfp* and *cat* reporter cassette fused to the promoter of *vp1400* (pT6SS1^report^), measured as OD_600_. Bacteria were grown at 30°C in M9 media supplemented with 3% (wt/vol) NaCl and kanamycin (250 µg/mL), to maintain the plasmids. Where indicated, the media were supplemented with phenamil (20 µM), DMSO (20% [vol/vol], added to the media as a control at the same volume of the phenamil solution), or the indicated concentrations of DOC or TDC (wt/vol). Data are shown as the mean ± SD, n = 3 independent replicates. The results from a representative experiment out of at least three independent experiments are shown. WT, wild-type.


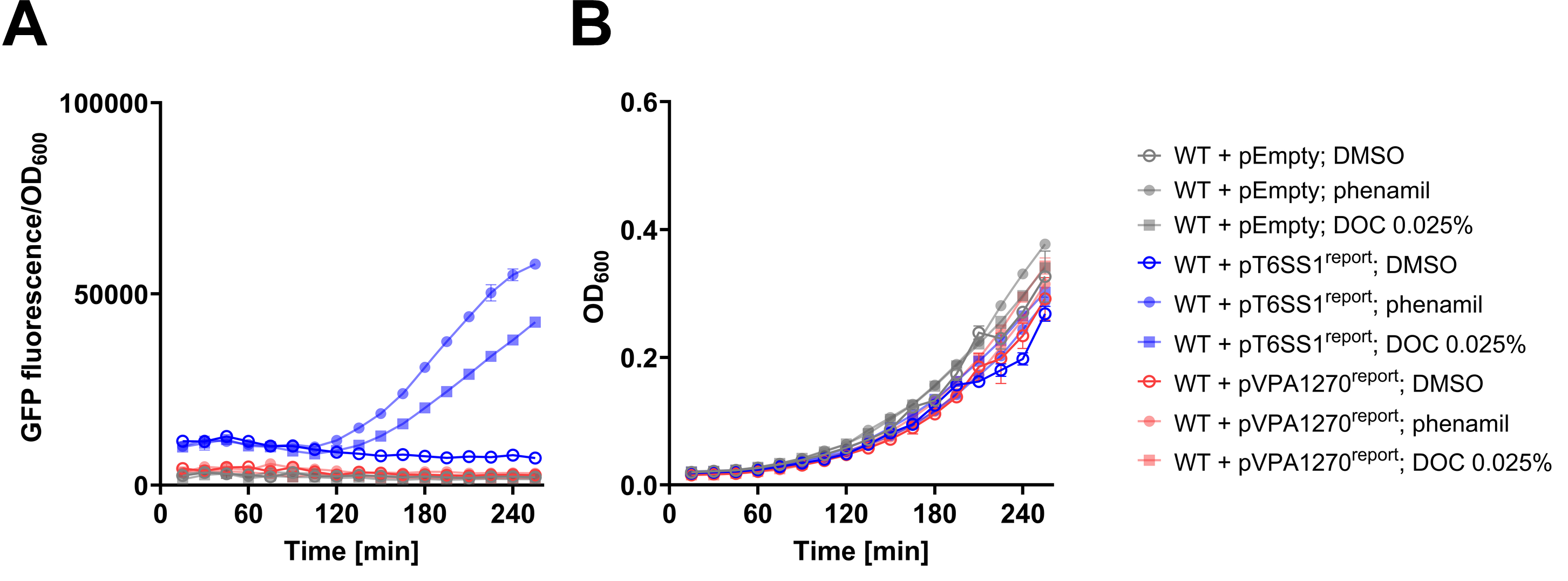


**Fig. S2. Bile acids and phenamil do not have a global effect on transcription and translation in *Vibrio parahaemolyticus*.** **A)** Fluorescence intensity measured as GFP fluorescence to OD_600_ (arbitrary units), and **B)** growth over time in *V. parahaemolyticus* RIMD 2210633 carrying an empty plasmid (pEmpty), a plasmid containing a *gfp* and *cat* reporter cassette fused to the promoter of *vp1400* (pT6SS1^report^), or a plasmid containing a *gfp* and *cat* reporter cassette fused to the promoter of *vpa1270* (pVPA1270^report^). Bacteria were grown at 30°C in M9 media supplemented with 3% (wt/vol) NaCl and kanamycin (250 µg/mL), to maintain the plasmids. Where indicated, the media were supplemented with phenamil (20 µM), DMSO (20% [vol/vol], added to the media as a control at the same volume of the phenamil solution), or DOC (0.025% [wt/vol]). Data are shown as the mean ± SD, n = 3 independent replicates. The results from a representative experiment out of at least three independent experiments are shown. WT, wild-type.

**Supplementary Tables**

**Table S1. A list of bacterial strains used in this study.**

| **Strain** | **Genotype** | **Comments** | **Source** |
| --- | --- | --- | --- |
| *Vibrio parahaemolyticus* RIMD 2210633 | Wild-type | Used for generating deletion strains, secretion assays, and in T6SS1 reporter assays | Obtained from Prof. Kim Orth; (6) |
| *Vibrio parahaemolyticus* RIMD 2210633 Δ*hns* | Δ*vp1133* | Used in T6SS1 reporter assays | (7) |
| *Vibrio parahaemolyticus* RIMD 2210633 Δ*tfoY* | Δ*vp1028* | Used in T6SS1 reporter assays | This study |
| *Vibrio parahaemolyticus* RIMD 2210633 Δ*hcp1* | Δ*vp1393* | Used in secretion assays | (8) |
| *Vibrio natriegens* ATCC 14048 | Wild-type | Used as prey in competition assays | ATCC collection |
| *Escherichia coli* DH5α (λ-pir) | K-12 derivative laboratory strain containing λ-pir | Used for plasmid maintenance and cloning | Obtained from Prof. Eric V. Stabb |

**Table S2. A list of plasmids used in this study.**

| **Plasmid** | **Description** | **Comments** | **Source** |
| --- | --- | --- | --- |
| pVSV33 | Kanamycin resistance; promoterless *cat* and *gfp* reporter operon | Parental plasmid for promoter investigation | Obtained from Prof. Eric V. Stabb; (1) |
| pT6SS1^report^ | pVSV33 containing the 300 bp upstream of the *vp1400* start codon transcriptionally fused to the GFP and chloramphenicol-resistance reporter cassette | Used to investigate expression from the *vp1400* promoter of *Vibrio parahaemolyticus* RIMD 2210633 | This study |
| pVPA1270^report^ | pVSV33 containing the 200 bp upstream of the *vpa1270* start codon transcriptionally fused to the GFP and chloramphenicol-resistance reporter cassette | Used to investigate expression from the *vpa1270* promoter of *Vibrio parahaemolyticus* RIMD 2210633 | This study |
| pDM4:*tfoY* | a Cm^R^ and ori_R6K_-containing suicide vector harboring 1 kb upstream and 1 kb downstream of *vp1028* in its MCS | Used to delete *tfoY* in *Vibrio parahaemolyticus* RIMD 2210633 | (9) |
| pBAD33.1 | pBAD series expression plasmid encoding the Cm^R^ gene and the p15A origin of replication | Used for selective growth of *V. natriegens* prey strains during competition assays | Obtained from Addgene (10) |

**Supplementary References**

1. Dunn AK, Millikan DS, Adin DM, Bose JL, Stabb E V. 2006. New *rfp*- and pES213-derived tools for analyzing symbiotic *Vibrio fischeri* reveal patterns of infection and *lux* expression in situ. Appl Environ Microbiol 72:802–810.

2. Gibson DG, Young L, Chuang RY, Venter JC, Hutchison CA, Smith HO. 2009. Enzymatic assembly of DNA molecules up to several hundred kilobases. Nat Methods 6:343–345.

3. Bensadoun A, Weinstein D. 1976. Assay of proteins in the presence of interfering materials. Anal Biochem 70:241–250.

4. Li P, Kinch LN, Ray A, Dalia AB, Cong Q, Nunan LM, Camilli A, Grishin N V, Salomon D, Orth K. 2017. Acute hepatopancreatic necrosis disease-causing Vibrio parahaemolyticus strains maintain an antibacterial type VI secretion system with versatile effector repertoires. Appl Environ Microbiol 83:e00737-17.

5. Salomon D, Gonzalez H, Updegraff BL, Orth K. 2013. Vibrio parahaemolyticus Type VI secretion system 1 Is activated in marine conditions to target bacteria, and is differentially regulated from system 2. PLoS One 8:e61086.

6. Makino K, Oshima K, Kurokawa K, Yokoyama K, Uda T, Tagomori K, Iijima Y, Najima M, Nakano M, Yamashita A, Kubota Y, Kimura S, Yasunaga T, Honda T, Shinagawa H, Hattori M, Iida T. 2003. Genome sequence of Vibrio parahaemolyticus: a pathogenic mechanism distinct from that of V cholerae. Lancet 361:743–749.

7. Dar Y, Jana B, Bosis E, Salomon D. 2022. A binary effector module secreted by a type VI secretion system. EMBO Rep 23:e53981.

8. Jana B, Fridman CM, Bosis E, Salomon D. 2019. A modular effector with a DNase domain and a marker for T6SS substrates. Nat Commun 10:3595.

9. Ben-Yaakov R, Salomon D. 2019. The regulatory network of Vibrio parahaemolyticus type VI secretion system 1. Environ Microbiol 21:2248–2260.

10. Chung HS, Raetz CRH. 2010. Interchangeable domains in the Kdo transferases of escherichia coli and haemophilus influenzae. Biochemistry 49:4126–4137.
